# Manganese-Enhanced Magnetic Resonance Imaging for Mapping of Whole Brain Activity Patterns Associated with the Intake of Snack Food in Ad Libitum Fed Rats

**DOI:** 10.1371/journal.pone.0055354

**Published:** 2013-02-07

**Authors:** Tobias Hoch, Silke Kreitz, Simone Gaffling, Monika Pischetsrieder, Andreas Hess

**Affiliations:** 1 Department of Chemistry and Pharmacy, Food Chemistry Division, Emil Fischer Center, University of Erlangen-Nuremberg, Erlangen, Germany; 2 Institute of Experimental and Clinical Pharmacology and Toxicology, Emil Fischer Center, University of Erlangen-Nuremberg, Erlangen, Germany; 3 Pattern Recognition Lab, University of Erlangen-Nuremberg, Erlangen, Germany; 4 School of Advanced Optical Technologies (SAOT), University of Erlangen-Nuremberg, Erlangen, Germany; Hosptial Infantil Universitario Niño Jesús, CIBEROBN, Spain

## Abstract

Non-homeostatic hyperphagia, which is a major contributor to obesity-related hyperalimentation, is associated with the diet’s molecular composition influencing, for example, the energy content. Thus, specific food items such as snack food may induce food intake independent from the state of satiety. To elucidate mechanisms how snack food may induce non-homeostatic food intake, it was tested if manganese-enhanced magnetic resonance imaging (MEMRI) was suitable for mapping the whole brain activity related to standard and snack food intake under normal behavioral situation. Application of the MnCl_2_ solution by osmotic pumps ensured that food intake was not significantly affected by the treatment. After z-score normalization and a non-affine three-dimensional registration to a rat brain atlas, significantly different grey values of 80 predefined brain structures were recorded in ad libitum fed rats after the intake of potato chips compared to standard chow at the group level. Ten of these areas had previously been connected to food intake, in particular to hyperphagia (e.g. dorsomedial hypothalamus or the anterior paraventricular thalamic nucleus) or to the satiety system (e.g. arcuate hypothalamic nucleus or solitary tract); 27 areas were related to reward/addiction including the core and shell of the nucleus accumbens, the ventral pallidum and the ventral striatum (caudate and putamen). Eleven areas associated to sleep displayed significantly reduced Mn^2+^-accumulation and six areas related to locomotor activity showed significantly increased Mn^2+^-accumulation after the intake of potato chips. The latter changes were associated with an observed significantly higher locomotor activity. Osmotic pump-assisted MEMRI proved to be a promising technique for functional mapping of whole brain activity patterns associated to nutritional intake under normal behavior.

## Introduction

Hyperphagia, which is associated with caloric hyperalimentation, substantially contributes to the development of obesity and obesity-related complications in industrial societies [1]. Whereas homeostatic hyperphagia is caused by a disturbance of the homeostatic system that regulates hunger and satiety, hedonic hyperphagia is rather independent from satiety [1]. The mechanisms, however, that override the physiological regulation of hunger and food intake are not fully elucidated. Under certain conditions, food intake may activate the brain reward system in a way that overcompensates the homeostatic control of appetite [2]. The resulting hedonic hyperphagia is influenced by several factors such as the consumer’s emotional state, mental health conditions or sleep deprivation [1]. Additionally, the molecular food composition and energy density seem to be important factors in the induction of hedonic hyperphagia. It is well documented that “palatable food” may induce hyperphagia in humans and animals [3], [4]. Binge eating episodes in humans, for example, often involve food rich in fats or sugars, or both [5].

Food intake in the state of hunger strongly triggers a complex reward system in the brain including the nucleus accumbens and ventral pallidum in the ventral striatum, the ventral tegmental area in the midbrain, the prefrontal cortex, the hippocampus and the amygdala [6]. These activation patterns are most likely associated with dopamine release, for example in the nucleus accumbens or dorsal striatum [7], [8], [9], processes which are also activated in drug addiction [10]. Under homeostatic conditions, however, satiety signals trigger brain structures such as the caudal brainstem, the hypothalamus, particularly the arcuate nucleus, or nucleus tractus solitarius, which limit food intake, for instance by decreasing its reward value [6], [11]. It had been observed that certain types of food, such as a high-fat or cafeteria diet, induce increased food and/or energy intake leading eventually to obesity. Ad libitum fed rats, for example, which had restricted access to a cafeteria diet, developed a binge-like feeding behavior during the access period [10]. Thus, it can be hypothesized that some food components can overrule the satiety regulation resulting in food ingestion independent from hunger.

Interestingly, it was shown that in mice, the initial fat-induced increase of food and calorie intake is compensated after a period of two weeks [12]. Thus, it was suggested that chronic intake of a high-fat diet decreases the rewarding effect of food, leading to disorganization of the feeding pattern which eventually results in overweight [13].

In order to cope with hedonic hyperphagia as a major contributor of obesity in industrial societies and its implications for the health care system, it is important to understand the cerebral processes that are triggered by certain types of food associated with hedonic binge-eating episodes. The application of non-invasive whole brain imaging techniques such as functional magnetic resonance imaging (MRI) for analyzing the influence of food intake on brain activity is limited in its classical, stimulus driven approach by the necessary synchronization of food intake and MRI. To monitor long-term effects on brain activity, manganese-enhanced MRI (MEMRI) has been employed. The contrast agent manganese accumulates in activated brain structures and reflects an integral measure of neuronal activity [14], [15], [16]. MEMRI allows the uncoupling of brain activity analysis from the MRI measurement. For this purpose, MnCl_2_ is injected prior to MRI measurement. Manganese ions (Mn^2+^) have a similar ionic radius and the same charge as calcium ions (Ca^2+^). Hence, Mn^2+^ is transported via voltage gated calcium channels into excitable cells. In contrast to Ca^2+^, however, Mn^2+^ accumulates in the cells proportionally to their activity and can be subsequently recorded by MRI due to its paramagnetic character. Thus, brain activity associated to events that took place up to several days before MRI measurement can be recorded. Therefore, the main advantage of this technique is the possibility to disentangle the stimulus (feeding) and the MRI measurement. Additionally, Mn^2+^ can be relocated by axonal transport to other brain areas. The major drawback of Mn^2+^, however, is its cytotoxicity, which may considerably influence natural behavior and limits the application in behavioral studies. It was shown that the subcutaneous injection of MnCl_2_ in concentrations sufficient for MRI analysis resulted in a persistent decrease in motor performance and food intake as well as in weight loss [17]. Recently, however, osmotic pumps were introduced to MEMRI studies. MnCl_2_ is administered by osmotic pumps, which slowly and continuously release the solution over a time period of up to seven days avoiding adverse effects on motor activity, but providing sufficient manganese accumulation for MRI analysis [17].

The present study tested the usability of osmotic pump-assisted MEMRI analysis to scan the whole brain activity associated with food uptake. The method was applied to unravel specific brain activation patterns of potato chip intake in ad libitum fed rats.

## Materials and Methods

### 1. Ethics Statement

This study was carried out in strict accordance with the recommendations of the Guide for the Care and Use of Laboratory Animals of the National Institutes of Health. The protocol was approved by the Committee on the Ethics of Animal Experiments of the University of Erlangen-Nuremberg (Regierung Mittelfranken, Permit Number: 54-2532.1-28/12). All surgery and MRI experiments were performed under isoflurane anesthesia, and all efforts were made to minimize suffering.

### 2. Experimental Design and Behavioral Analysis

**Figure 1:**
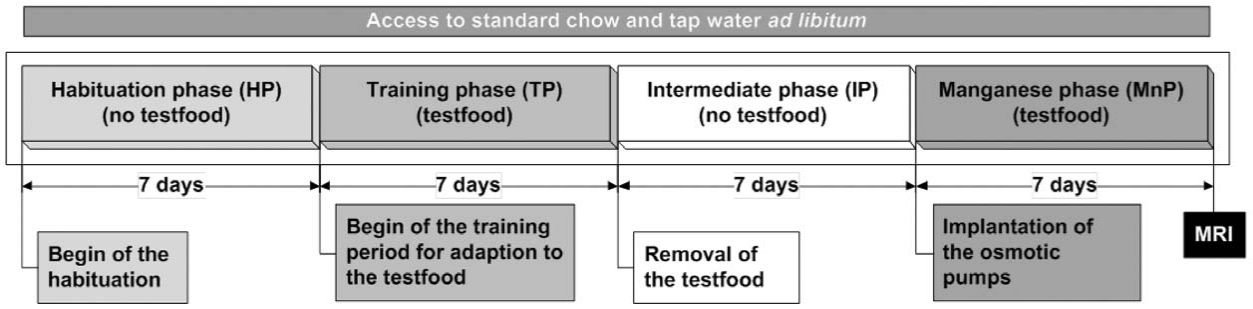
Study design. Overview on the study design for monitoring the influence of food composition on whole brain activity patterns by manganese-enhanced magnetic resonance imaging.

Male Wistar rats (initial weight 257±21 g, kept in a 12/12 h dark/light cycle, purchased from Charles River, Sulzfeld, Germany) were randomly divided into two groups (four cages per group, four animals per cage). Each group received one of the different foods additional to their standard chow pellets (Altromin 1326, Altromin, Lage, Germany). The snack food group (n = 16, initial body weight 258±28 g) received potato chips (commercial unflavored salted potato chips without added taste compounds or taste enhancer, particularly no monosodium glutamate, crushed by a food processor) and the standard chow group (initial body weight 256±21 g) received powdered standard chow (Altromin 1321, n = 16), respectively. Standard chow pellets were offered ad libitum over the whole course of the study, the test food (crushed potato chips or powdered standard chow, respectively), was offered ad libitum during the training phase and the manganese phase additionally to standard chow pellets (see **Figure 1** for experimental design). For training, the test foods were presented in two food dispensers containing identical test food on the right and the left side of the cage over a period of seven days (training phase), followed by seven intermediate days (intermediate phase) without test foods. Subsequently, osmotic pumps filled with manganese chloride (MnCl_2_, see below for details) were implanted. Over the period of the drip injection (seven days, standard chow group: 163±5 h, snack food group 166±4 h) and accumulation of MnCl_2_ in the rat brain (manganese phase) the animals had ad libitum access to the test food familiar from the training phase. Since the standard chow pellets and tap water were available ad libitum during all phases of the study, animals were not fasted at any time during the study. The active brain structures were scanned by MEMRI after this period of MnCl_2_ administration_._ During the different phases, the amount of ingested food was measured by differential weighing of the food dispensers twice a day. The energy intake was determined by multiplying the caloric values of the test foods with the ingested amounts. The food intake correlated positively with the initial body weight of the rats. However, the correlation was similar for both types of test food and the distribution of initial body weight did not differ significantly between both groups.

Additionally, the locomotor activity associated with the test foods was quantified by the evaluation of pictures recorded by webcams above the cages (one picture per ten seconds) via defined “counts”. One “count” was defined as “one rat shows locomotor activity near the food dispensers on one picture”. The student t-test was used to evaluate significant differences in the locomotor activity of the rats in the different groups during 24 h per day with one-hour bins over seven days as a mean of four cages (16 animals) per group.

### 3. Preparation and Implantation of the Osmotic Pumps

Mini-osmotic pumps (Alzet®, model 2001, Durect Corporation, Cupertino, CA, USA) were used for the application of the contrast agent (200 µL of a 1 M solution of MnCl_2_, for molecular biology, BioReagent, Sigma Aldrich, Schnelldorf, Germany) according to [17]. For the use in MRI, the stainless steel flow moderator was replaced by a PEEK™ micro medical tubing (Scientific Commodities, Lake Havasu City, AZ, USA). The filled osmotic pumps were incubated in isotonic saline for 12 h previous to implantation. During the seven days drip injection, MnCl_2_ was released with a flow rate of 1 µL h^−1^.

In the afternoon of the first day of the manganese phase (see **Figure 1**), osmotic pumps were implanted. For this purpose, the animals were anesthetized for a maximal time of 15 minutes with isoflurane (initially 5% and 1.5% maintenance, Baxter Deutschland, Unterschleißheim, Germany) in medical air and the filled pumps were implanted in dorsal subcutaneous tissue. Afterwards, the small cut was closed by tissue glue (Histoacryl®, B. Braun Petzold, Melsungen, Germany).

### 4. MRI Measurement

After seven days of the manganese phase, MRIs were recorded. The animals were anesthetized with isoflurane (initially 5% in medical air) 163±5 h (standard chow group) and 166±4 h (snack food group) after the implantation of the osmotic pumps. Anesthesia lasted for a maximum of 50 minutes for each animal. After anesthesia induction, animals were placed on a cradle inside the magnetic resonance tomograph (Bruker BioSpec 47/40, 200 mT/m, quadrature surface brain coil). Body temperature of the animals was kept constant at 37°C by warm water circulating in the cradle. The fixation of the rat’s head and continuous isoflurane anesthesia were ensured by a “nose-mouth mask” directly below the surface coil. Vital functions of the animals were monitored during the measurement via a breathing sensor fixed under the chest of the rat. To keep the respiration rate constant at about 60 min^−1^, the isoflurane concentration was adjusted in a range between 1% and 2%.

The measurement was carried out using a modified driven equilibrium Fourier transform (MDEFT) sequence: repetition time 4 s, echo time 5.2 ms, inversion time 1000 ms, with four segments and an acquisition matrix of 256×128×32, reconstruction matrix after zero filling 256×256×64 with a resolution of 109×109×440 µm, field of view 27.90×27.90×28.16 mm and two averages resulting in a measurement time of 17 min repeated twice.

### 5. Data Processing

#### 5.1 Image registration and preprocessing

To investigate differences in brain anatomy/function, all data sets had to be transferred into a common coordinate system. The goal was to match the anatomy without eliminating the relevant differences. This was achieved using a non-parametric, non-rigid registration scheme, which calculated a deformation field for a template volume T, indicating a translation vector for each voxel in such a way that the similarity of the deformed template volume to the reference volume R was maximal.

The registration method optimized an energy functional consisting of a data term measuring the similarity of the two data sets under the current transform (here mutual information), and a regularization term restricting the allowed deformation. In our case, the smoothness of the deformation was assured by regularization of the curvature of the deformation field, as introduced in [18]. Registration was done using a custom implementation of the employed non-rigid registration components [19].

First, all data sets belonging to one group were non-rigidly registered onto a randomly chosen reference volume of that group, and the group-wise average volume and a variance volume were calculated. Afterwards, all group-wise average volumes were subsequently non-rigidly registered to one of the volumes, and the respective deformation field applied to the group-wise variance volume. Finally, an overall average volume and variance volume was calculated. By voxel-based morphometric analysis (VBM), significantly (t-statistics) different activated brain areas between the two food groups could be determined. Using voxelwise statistics on the registered data sets also allowed cancelling basic tissue contrasts in the images, which were the same in both groups.

#### 5.2 Grey value processing for structure-specific analysis

The grey value analysis based on these preregistered data sets was performed in MagnAN (BioCom GbR, Uttenreuth, Germany). A surface-based registration adjusted each MEMRI grey value dataset to the digital rat brain atlas derived from [20]. Next, to compensate for minor individual shape differences, the atlas slides were fine-adjusted slice by slice for each dataset guided by the outlines of the brain and the ventricular system. The digital atlas consisted of 166 preselected distinct brain structures. The ventral tegmental area (VTA) is one of the smallest structures evaluated, but has high impact on the obtained results. It has a volume of 0.7914 mm^3^ per hemisphere, i.e. 152 voxels. In each spatial dimension, the VTA was sampled with more than 4 voxels. Therefore, partial volume effects, which could cause major confounding problems in our analysis, could be avoided. The mean grey values of these regions were determined on the individual data sets. For normalization of the grey values of each individual, z-scores were calculated by dividing the difference between the grey value of every single brain structure and the mean grey value of all atlas structures by the standard deviation of the grey values of all atlas structures. The student t-test was used to evaluate significant differences of the brain structures between the two different groups. The combined analysis approach allowed obtaining the significant different areas (VBM) as well as the activity up- and downregulation within the corresponding atlas regions (region based).

## Results and Discussion

### 1. Effect of Snack Food (Potato Chips) Diet on Calorie Intake and Locomotor Activity

The present study investigated specific brain activity patterns related to the intake of snack food (potato chips) compared to standard chow. Brain activity related to the intake of the particular test food was recorded by MEMRI, which allowed integrating the brain activity over the period of seven days of food intake **(** **Figure 1** **)**.

**Figure 2:**
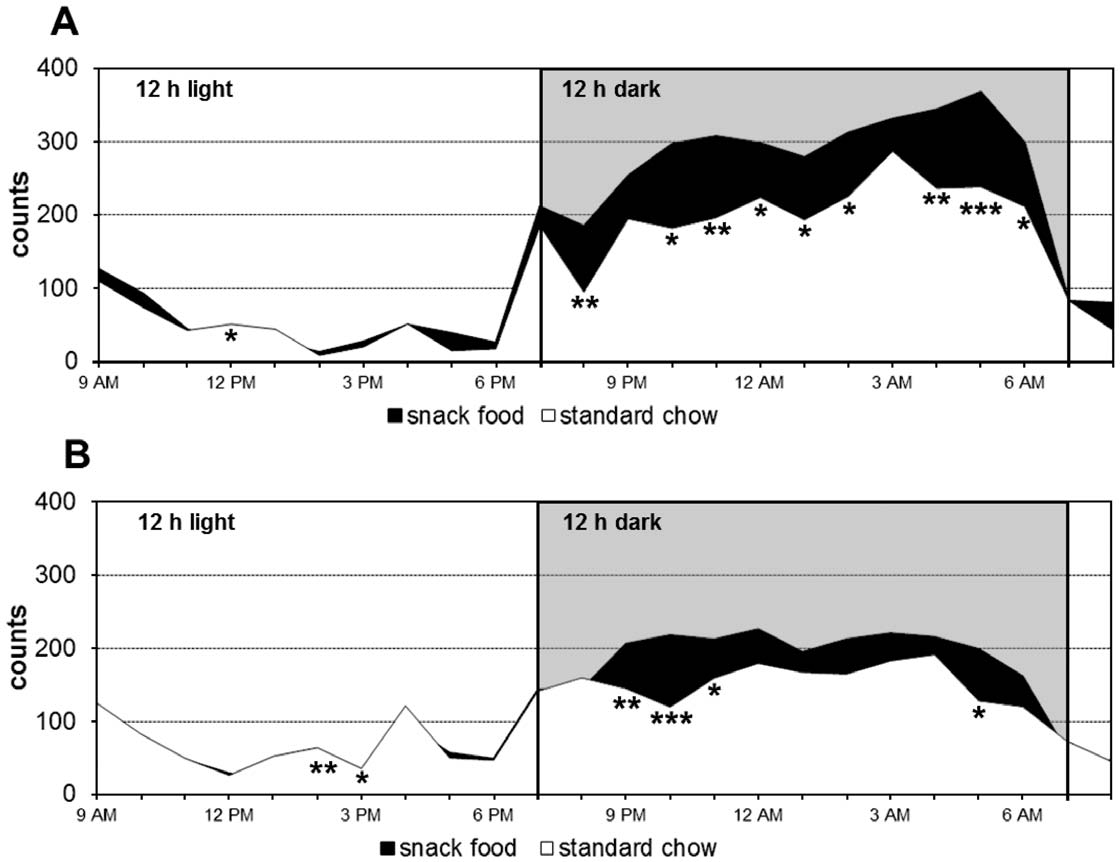
Feeding-related locomotor activity during access to snack food (potato chips) or standard chow. Feeding-related locomotor activity of the rats during access to snack food (potato chips) or standard chow in the training phase (A) and manganese phase during MnCl_2_ application (B). Data are presented as the mean of 16 animals over 7 d per group. ***p<0.001, **p<0.01, p*<0.05.

Additionally, food intake and locomotor activity dependent on the test food were recorded. During the training phase, rats fed with standard chow showed continuously lower activity than rats fed with potato chips, especially in the dark period of the 12/12 h dark/light cycle. Potato chip intake induced higher activity with significant differences at 10 out of 24 time points in the training phase **(** **Figure 2A** **)**.

### 2. Application of Osmotic Pump-assisted MEMRI for the Analysis of Diet-associated Whole Brain Activity Patterns

For the analysis of active brain patterns, osmotic pump assisted MEMRI was applied. Whereas a single dose of MnCl_2_ led to a maximal accumulation 24 h after injection, manganese accumulation in the brain via osmotic pumps reached a plateau after three days [17]. The obtained cumulative concentration of Mn^2+^ was sufficient for functional mapping resulting in a similar signal-to-noise ratio as obtained by a single-dose injection of MnCl_2_, but the motor activity was not affected under these conditions [17]. Differences in general Mn^2+^ distribution due to different permeability of brain structures to Mn^2+^ should be the same in both groups. Z-Score differences between the groups were used to evaluate test food-related brain activity instead of absolute z-score values. Consequently, brain areas which had been active during the seven-day period of the manganese phase could be recorded by a single MRI measurement **(** **Figure 1** **)**. In our case, osmotic pump assisted MEMRI rendered a comprehensive view of test food-induced whole brain activity.

**Figure 3:**
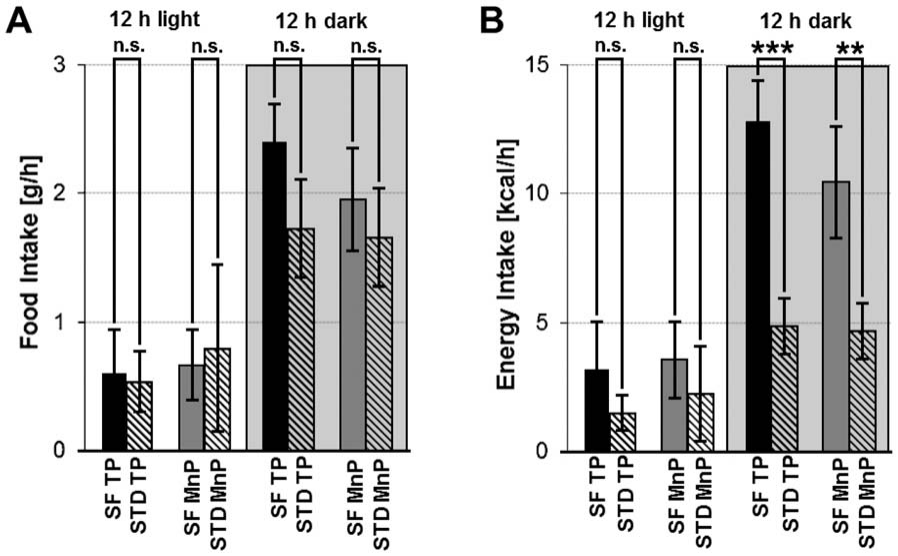
Food and energy intake via snack food (potato chips) and standard chow. Food (A) and energy (B) intake via snack food (SF, potato chips) and standard chow (STD) in ad libitum fed rats in the training phase (TP) before and in the manganese phase (MnP) during MnCl_2_ pump infiltration over a period of 7 d. Food intake per hour was determined by differential weighing, energy intake by multiplying the amount of the ingested food with the energy content separately during the 12 h light and the 12 h dark cycle. The mean ± SD of 16 animals in each group is shown. ***p<0.001, **p<0.01, p*<0.05, n.s. not significant.

The present study recorded a somewhat reduced total motor activity during the manganese phase compared to the training phase **(** **Figure 2B** **)**. This may be due to the implantation and the associated stress, the cytotoxicity of the manganese or to habituation effects concerning the test food. Nevertheless, rats fed with potato chips displayed clearly higher activity compared to the control with significantly increased activity at four time points. This behavior was similar to the training phase. Otherwise, the amount of ingested food was not significantly altered during the manganese phase compared to the training phase regarding both the 12 h light and the 12 h dark cycle. A slightly increased intake of the snack food during the 12 h dark cycle compared to the standard chow both in the training and the manganese phase was detected **(** **Figure 3A** **)**. This led to a higher energy intake through potato chips compared to standard chow. The difference was not significant during the 12 h light period, but highly significant during the 12 h dark period both during training phase and manganese phase **(** **Figure 3B** **)**. Thus, it was concluded that MnCl_2_ administration by osmotic pumps is a suitable method for mapping activity patterns in the brain specific for different ingested foods.

**Figure 4:**
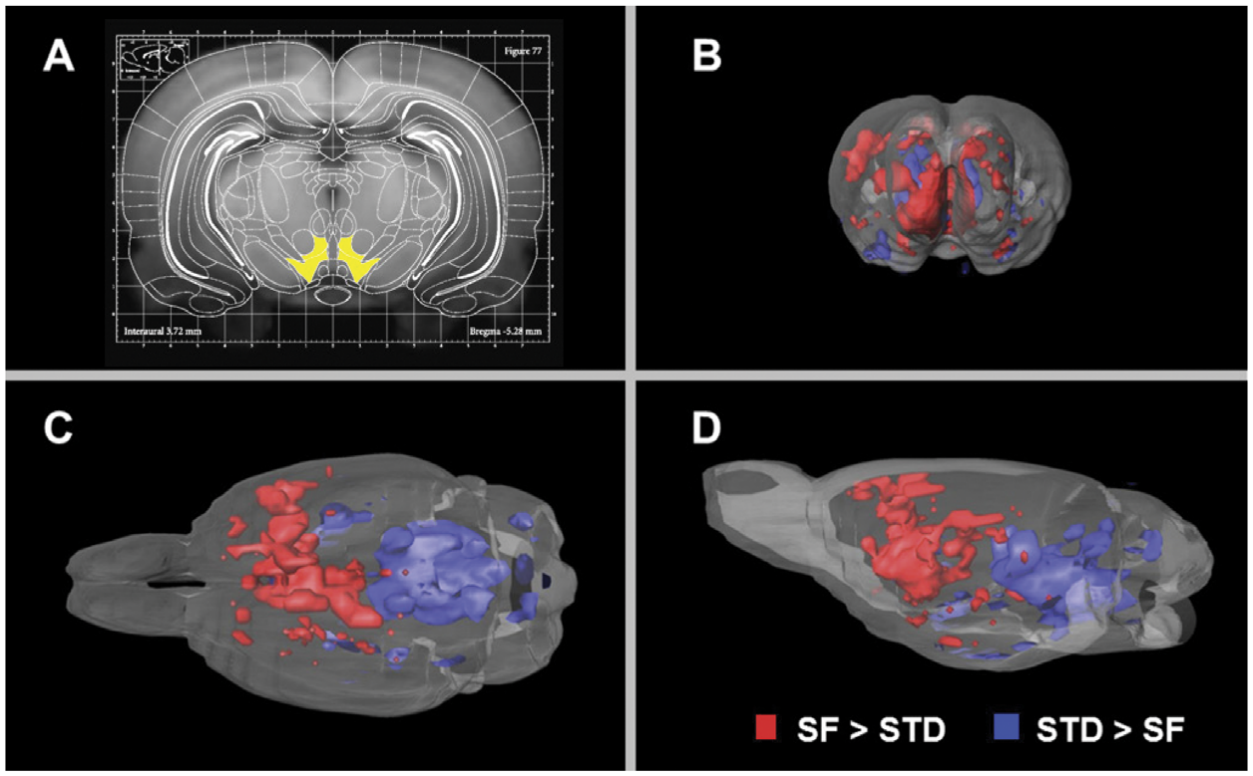
Significantly different manganese accumulation in the brain in relation to standard chow or snack food (potato chips). In (A) the overlay of a slice of the reconstructed average modified driven equilibrium Fourier transform (MDEFT) dataset with the corresponding atlas slice (Bregma −5.28 mm) from the Paxinos atlas is shown with one of the smallest analyzed regions (VTA) marked in yellow. Parts (B), (C) and (D) show the significantly different manganese accumulation in the brain of ad libitum fed rats with additional access to standard chow (STD) or snack food (SF, potato chips) recorded by MEMRI. Brain areas with significantly higher activity due to the intake of snack food compared to the intake of standard chow are marked in red, brain areas which showed a significantly higher activity after the intake of standard chow compared to the intake of snack food are marked in blue. Data were processed by voxelwise statistical analysis. The results are displayed in axial (B), horizontal (C) and sagital (D) view.

After z-score normalization, image data were analyzed on the one hand by a VBM approach, which resulted – purely data driven - in significantly differently activated brain areas **(** **Figure 4** **)**. On the other hand, the additional region-based analysis using a digital atlas made it possible to determine up- and downregulations of each labeled atlas structure.

**Figure 5:**
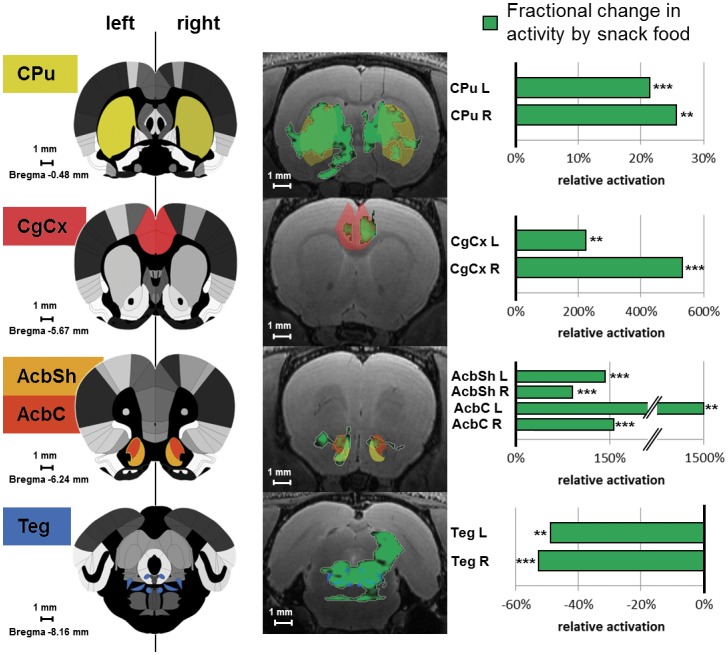
Activation differences related to snack food (potato chips) vs. standard chow in representative brain structures. Statistics of activation differences due to the intake of snack food (potato chips) vs. standard chow in representative brain structures for the motor circuit (caudate putamen: CPu), the limbic system (cingulate cortex: CgCx), the reward system (shell region of the nucleus accumbens: AcbSh, core region of the nucleus accumbens: AcbC) and sleep/wake rhythm (tegmental nuclei: Teg) depicted in the left column based on the reference atlas. The middle column shows significant differences of the VBM analysis overlaid on corresponding standard T2 weighted MRI anatomy and atlas labels. The right column shows the fractional change of snack food to standard chow v (MEMRI grey values) ***p<0.001, **p<0.01.

Significantly different z-scores were detected in 80 brain areas when standard chow and snack food (potato chips) were compared **(** **Tables 1** **,** **2** **,** **3** **,** **4** **)**. In general, both different data-analysis strategies led to comparable results. Differential MEMRI activation of the most relevant brain structures after the intake of potato chips compared to standard chow is depicted for selected brain structures (**Figure 5** **)**.

**Table 1:** Manganese accumulation in brain structures related to food intake.

Brain structure	z-Score ± SD		p-Value
	Standard chow	Snack food	
Arcuate hypothalamic nucleus L	−2.33±0.25	−2.48±0.30	0.0383 (*)
Dorsomedial hypothalamus R	−2.00±0.31	−1.71±0.52	0.0109 (*)
Infralimbic cortex L	−1.50±0.46	−1.00±0.60	0.0005 (***)
Infralimbic cortex R	−1.58±0.47	−1.10±0.62	0.0014 (**)
Lateral hypothalamus R	−0.29±0.23	−0.15±0.21	0.0208 (*)
Paraventricular thalamic nucleus anterior	−1.04±0.19	−0.69±0.56	0.0024 (**)
Raphe nucleus	0.31±0.27	−0.06±0.25	0.0000 (***)
Septum L	0.03±0.49	0.55±0.49	0.0001 (***)
Septum R	−0.21±0.44	0.39±0.53	0.0000 (***)
Solitary tract R	−0.86±0.27	−1.08±0.31	0.0054 (**)

Significantly different (***p<0.001, **p<0.01, p*<0.05) manganese accumulation in brain structures of ad libitum fed rats with additional access to standard chow or snack food (potato chips) recorded by MEMRI. Data were processed by region-based analysis of distinct brain structures after z-scores normalization. SD, standard deviation; L, left side; R, right side.

**Table 2:** Manganese accumulation in brain structures related to reward and addiction.

Brain structure	z-Score ± SD		p-Value
	Standard chow	Snack food	
Arcuate hypothalamic nucleus L	−2.33±0.25	−2.48±0.30	0.0383 (*)
Bed nucleus of stria terminalis L	0.68±0.38	1.16±0.30	0.0000 (***)
Caudate putamen (striatum) L	1.73±0.44	2.10±0.30	0.0003 (***)
Caudate putamen (striatum) R	1.29±0.40	1.62±0.40	0.0021 (**)
Cingulate cortex L	−0.18±0.54	0.22±0.46	0.0025 (**)
Cingulate cortex R	0.11±0.65	0.69±0.52	0.0003 (***)
Dorsal subiculum L	−0.98±0.53	−0.50±0.75	0.0059 (**)
Dorsal subiculum R	−1.22±0.44	−0.83±0.55	0.0031 (**)
Insular cortex (insula) L	−0.94±0.27	−0.74±0.28	0.0071 (**)
Insular cortex (insula) R	−1.28±0.25	−1.09±0.30	0.0118 (*)
Interpeduncular nucleus	−0.70±0.39	−1.12±0.96	0.0331 (*)
Lateral parabrachial nucleus R	0.12±0.40	0.14±0.53	0.0356 (*)
Mediodorsal thalamic L	0.33±0.31	0.60±0.39	0.0039 (**)
Mediodorsal thalamic R	0.22±0.19	0.46±0.28	0.0002 (***)
Nucleus accumbens (core subregion) L	0.03±0.30	0.41±0.49	0.0005 (***)
Nucleus accumbens (core subregion) R	−0.28±0.31	0.16±0.53	0.0003 (***)
Nucleus accumbens (shell subregion) L	−0.30±0.33	0.13±0.51	0.0003 (***)
Nucleus accumbens (shell subregion) R	−0.56±0.34	−0.05±0.57	0.0001 (***)
Prelimbic cortex L	−1.11±0.27	−0.73±0.47	0.0004 (***)
Prelimbic cortex R	−1.06±0.41	−0.65±0.56	0.0024 (**)
Raphe nucleus	0.31±0.27	−0.06±0.25	0.0000 (***)
Ventral pallidum L	1.28±0.53	1.58±0.34	0.0096 (**)
Ventral pallidum R	1.01±0.42	1.25±0.41	0.0242 (*)
Ventral subiculum L	−2.48±0.36	−2.71±0.35	0.0114 (*)
Ventral subiculum R	−2.66±0.32	−2.86±0.26	0.0112 (*)
Ventral tegmental area L	0.84±0.26	0.59±0.22	0.0001 (***)
Ventral tegmental area R	0.73±0.25	0.53±0.21	0.0014 (**)

Significantly different (***p<0.001, **p<0.01, p*<0.05) manganese accumulation in brain structures of ad libitum fed rats with additional access to standard chow or snack food (potato chips) recorded by MEMRI. Data were processed by region-based analysis of distinct brain structures after z-scores normalization. SD, standard deviation; L, left side; R, right side.

**Table 3:** Manganese accumulation in brain structures related to sleep.

Brain structure	z-Score ± SD		p-Value
	Standard chow	Snack food	
Gigantocellular reticular nucleus L	−0.33±0.32	−0.64±0.28	0.0002 (***)
Gigantocellular reticular nucleus R	−0.36±0.32	−0.66±0.26	0.0001 (***)
Lateral paragigantocellular nucleus L	−0.94±0.29	−1.20±0.23	0.0002 (***)
Lateral paragigantocellular nucleus R	−0.97±0.27	−1.24±0.20	0.0000 (***)
Lateral reticular nucleus R	−1.03±0.35	−1.21±0.35	0.0411 (*)
Parvicellular reticular nucleus L	−0.21±0.32	−0.51±0.31	0.0004 (***)
Parvicellular reticular nucleus R	−0.29±0.29	−0.59±0.25	0.0001 (***)
Pontine reticular nucleus oral L	0.77±0.35	0.39±0.32	0.0000 (***)
Pontine reticular nucleus oral R	0.72±0.36	0.33±0.28	0.0000 (***)
Tegmental nuclei L	0.64±0.36	0.33±0.31	0.0006 (***)
Tegmental nuclei R	0.58±0.30	0.27±0.31	0.0002 (***)

Significantly different (***p<0.001, **p<0.01, p*<0.05) manganese accumulation in brain structures of ad libitum fed rats with additional access to standard chow or snack food (potato chips) recorded by MEMRI. Data were processed by region-based analysis of distinct brain structures after z-scores normalization. SD, standard deviation; L, left side; R, right side.

**Table 4:** Manganese accumulation in brain structures related to locomotor activity.

Brain structure	z-Score ± SD		p-Value
	Standardchow	Snackfood	
Caudate putamen (striatum) L	1.73±0.44	2.10±0.30	0.0003 (***)
Caudate putamen (striatum) R	1.29±0.40	1.62±0.40	0.0021 (**)
Primary motor cortex L	1.32±0.56	1.64±0.36	0.0076 (**)
Primary motor cortex R	1.06±0.69	1.43±0.54	0.0230 (*)
Secondary motor cortex L	0.38±0.52	0.63±0.44	0.0436 (*)
Secondary motor cortex R	0.32±0.66	0.67±0.56	0.0307 (*)

Significantly different (***p<0.001, **p<0.01, p*<0.05) manganese accumulation in brain structures of ad libitum fed rats with additional access to standard chow or snack food (potato chips) recorded by MEMRI. Data were processed by region-based analysis of distinct brain structures after z-scores normalization. SD, standard deviation; L, left side; R, right side.

The achieved final registration quality is depicted in **Figure 4A** and **Figure 5**.

### 3. Influence of Snack Food (Potato Chips) Intake on Reward and Satiety Circuits

In the present study, the ingestion of potato chips led to a variety of different structure-specific activity changes, which are summarized in **Tables 1** **,** **2** **,** **3** **,** **4**. Significantly increased activity was found for the core and shell of nucleus accumbens (right and left side (R+L)), the ventral globus pallidus (R+L), and the dorsomedial hypothalamus (R) and the anterior paraventricular thalamic nucleus. At the same time, the arcuate nucleus (L) and the nucleus tractus solitarius (R), were deactivated in rats that ingested potato chips compared to animals fed on standard chow. Central mechanisms regulating food intake and appetite were recently summarized by Harrold et al. and Kenny [4], [21]: homeostatic regulation of food intake is mainly induced by signals reflecting an energy deficit [21]. Hedonic food intake, in contrast, seems to be driven by the activation of reward mechanisms overcompensating homeostatic downregulation of food intake [21].

The nucleus tractus solitarius is responsible for processing peripheral signals that reflect ongoing food intake, such as gastric distension or portal-vein glucose levels resulting in the deactivation of brain areas, such as the nucleus accumbens, eventually leading to a downregulation of energy intake [4], [22]. Inactivation of the nucleus tractus solitarius by “palatable food” may be mediated by a decreased sensitivity of this brain area towards satiety-related gut hormones [4]. Similar to the nucleus tractus solitarius, the arcuate hypothalamic nucleus is activated by peripheral signals reflecting the nutritional status. It is connected to other brain regions, such as the paraventral nucleus and the dorsomedial hypothalamic nucleus, which both control food intake [21], [23], [24]. Thus, it can be assumed that the activity changes of the nucleus tractus solitarius, the arcuate nucleus, the dorsomedial hypothalamus and the paraventrical thalamic nucleus anterior, which were observed in this study, reflect a deactivation of central satiety circuits, which eventually results in a calorie intake exceeding the energy need.

Additionally, strong activation of the nucleus accumbens related to potato chip intake has been observed. The nucleus accumbens is a key structure of the reward system, which is activated, for example, by rewarding drugs [9]. In the context of food intake, activation of the nucleus accumbens results in a rewarding signal inducing hedonic food intake. Additionally, a significantly increased activation upon consumption of potato chips was recorded in areas previously attributed to the general reward systems or addiction, namely the prelimbic cortex (R+L) [25], [26], the dorsal subiculum (R+L) [27], the bed nuclei of stria terminalis (L) [28], mediodorsal thalamus (R+L) [26], [29], the cingulate cortex (R+L) [26], caudate/putamen (ventral striatum) (R+L) [26] and the insular cortex (R+L) [30]. Mediodorsal thalamus and insular cortex have also been associated to olfaction or the integration of an olfactory with other sensory input [31]. Caudate and insula are also associated to drug- as well as food craving [32]. Further brain structures, which have been associated with reward and addiction, showed a significantly lower activity after the intake of snack food compared to standard chow: the raphe [33], the interpeduncular nucleus [34], the ventral tegmental area (R+L) [35], [36], and the ventral subiculum (R+L) [37].

These results indicate that consumption of potato chips is related to activation of hedonic reward circuits and, in parallel, to inactivation of homeostatic satiety circuits. Both circuits are also linked, mainly by the paraventricular nucleus of the thalamus, which acts as an interface between energy balance and reward [38]. Thus, the observed activation pattern may result in higher energy intake when snack food, such as potato chips, is available.

Further studies are now required to reveal the molecular components of potato chips, the role of the energy density as well as peripheral and central mechanisms that lead to a disregulation of the homeostatic control of energy uptake.

### 4. Influence of Snack Food (Potato Chips) Intake on other Brain Structures Related to Food Intake

Furthermore, after the consumption of snack food (potato chips), a stronger activation of those brain structures was observed that have previously been associated with food intake, appetite behavior and food control, such as the infralimbic cortex (R+L) [36], [39], the lateral hypothalamus (R) [36], and the septum (R+L) [40].

The brain structures raphe nuclei and lateral parabrachial nucleus (R), which have also been connected to food intake, showed significantly reduced activity after the consumption of potato chips compared to standard chow [41]. The lateral parabrachial nucleus has been associated with caloric regulation, ingestive reward, cognitive processing in feeding [42], but also with sodium and water intake [43]. Thus, the reduced activity of this structure may be associated with the higher salt content of the potato chips compared to standard chow. The results indicate that, due to its molecular composition, which results for example in a higher energy density, potato chips may activate brain structures associated with reward and the control of food intake differently than standard chow. This effect may eventually modulate the quality and quantity of food or rather energy intake.

### 5. Influence of Snack Food (Potato Chips) Intake on Brain Structures Related to Locomotor Activity and Sleep

Additionally, six brain structures connected to movement and activity showed significantly higher Mn^2+^ accumulation when rats had access to potato chips compared to standard chow: the primary motor cortex (R+L), the secondary motor cortex (R+L) as well as the caudate putamen (R+L) [44]. Significantly elevated activity of motor areas in the animals fed with potato chips is in good agreement with the behavioral studies, which show higher locomotor activity in this group **(** **Figure 2A and B** **)**. Increase of locomotor activity has been linked before with food intake. Thus, it was shown, for example, that ghrelin induced the intake of rewarding food as well as locomotor activity in rodents, which is probably related to the stimulation of food-seeking behavior [**45**], [**46**].

Finally, the ingestion of potato chips was connected with a significant deactivation of brain structures related to sleep, namely the lateral reticular nucleus (R) [47], the parvicellular reticular nucleus (R+L) [47], the lateral paragigantocellular nucleus (R+L) [48], the gigantocellular nucleus (R+L) [49], [50], the pontine reticular nucleus oral (R+L) [51] and the tegmental nuclei (R+L) [52]. The influence of food composition on sleeping behavior is not fully understood. It has been shown that a long-term (six weeks) intake of a high-fat diet led to an increase of frequency and duration of sleeping episodes. This effect, however, was rather related to the developing obesity than to the energy intake itself [53]. On the other hand, several studies revealed that a long-term application of a high-fat diet induces increased food intake during the diurnal resting period in mice [12], [54]. Increased diurnal food intake is most likely related to changes of sleeping behavior and consequently to modulation of brain-structure activity related to sleep. Under the short-term feeding conditions applied here, however, snack food induced neither a significant increase of body weight nor a shift of the circadian feeding pattern. Therefore, we speculate that the deactivation of sleep-related brain structures is linked to the increase of locomotor and food seeking activity, which may suppress sleep.

### Conclusions

In summary, MEMRI and the subsequent analysis of activated brain structures by both VBM as well as region-of-interest-based approach showed similar specific activation resp. deactivation of numerous brain structures dependent on the ingested food. Intake of snack food (potato chips) compared to standard chow by ad libitum fed rats induced significant differences in the activation patterns in brain structures that had been associated before with food intake, reward/addiction, as well as activity and movement. Increases in the cerebral locomotor activity structures were in accordance with the animal behavior: activity profiles over several days showed that a higher level of locomotor activity of the animals was associated with the intake of potato chips. Reduced activity was recorded in brain structures that are important for the regulation of the sleep-wake-rhythm, especially of REM-sleep.

The observed changes of brain activity patterns related to food intake are probably caused by the molecular composition of the snack food, resulting, for example, in a higher energy density. Additionally, the calorie supply by the snack food may induce modulation of brain activity patterns. Further studies are now required to reveal the triggers of the observed changes either by introducing a snack food group with control-matched calorie intake or by testing the effects of defined snack food components on brain activity patterns.
